# Genome3D: A viewer-model framework for integrating and visualizing multi-scale epigenomic information within a three-dimensional genome

**DOI:** 10.1186/1471-2105-11-444

**Published:** 2010-09-02

**Authors:** Thomas M Asbury, Matt Mitman, Jijun Tang, W Jim Zheng

**Affiliations:** 1 Department of Biochemistry & Molecular Biology, Medical University of South Carolina, 135 Cannon Street, Suite 303E, Charleston SC 29425, USA; 2 Department of Computer Science and Engineering, Swearingen Center, Room 3A61, University of South Carolina, Columbia, SC, 29208, USA

## Abstract

**Background:**

New technologies are enabling the measurement of many types of genomic and epigenomic information at scales ranging from the atomic to nuclear. Much of this new data is increasingly structural in nature, and is often difficult to coordinate with other data sets. There is a legitimate need for integrating and visualizing these disparate data sets to reveal structural relationships not apparent when looking at these data in isolation.

**Results:**

We have applied object-oriented technology to develop a downloadable visualization tool, *Genome3D*, for integrating and displaying epigenomic data within a prescribed three-dimensional physical model of the human genome. In order to integrate and visualize large volume of data, novel statistical and mathematical approaches have been developed to reduce the size of the data. To our knowledge, this is the first such tool developed that can visualize human genome in three-dimension. We describe here the major features of *Genome3D *and discuss our multi-scale data framework using a representative basic physical model. We then demonstrate many of the issues and benefits of multi-resolution data integration.

**Conclusions:**

*Genome3D *is a software visualization tool that explores a wide range of structural genomic and epigenetic data. Data from various sources of differing scales can be integrated within a hierarchical framework that is easily adapted to new developments concerning the structure of the physical genome. In addition, our tool has a simple annotation mechanism to incorporate non-structural information. *Genome3D *is unique is its ability to manipulate large amounts of multi-resolution data from diverse sources to uncover complex and new structural relationships within the genome.

## Background

A significant portion of genomic data that is currently being generated extends beyond traditional primary sequence information. Genome-wide epigenetic characteristics such as DNA and histone modifications, nucleosome distributions, along with transcriptional and replication center structural insights are rapidly changing the way the genome is understood. Indeed, these new data from high-throughput sources are often demonstrating that much of the genome's functional landscape resides in extra-sequential properties.

With this influx of new detail about the higher-level structure and dynamics of the genome, new techniques will be required to visualize and model the full extent of genomic interactions and function. Genome browsers, such as the USCS Genome Database Browser [1], are specifically aimed at viewing primary sequence information. Although supplemental information can easily be annotated via new tracks, representing structural hierarchies and interactions is quite difficult, particularly across non-contiguous genomic segments [2]. In addition, in spite of the many recent efforts to measure and model the genome structure at various resolutions and detail [3-10], little work has focused on combining these models into a plausible aggregate, or has taken advantage of the large amount of genomic and epigenomic data available from new high-throughput approaches.

To address these issues, we have created an interactive 3D viewer, *Genome3D*, to enable integration and visualization of genomic and epigenomic data. The viewer is designed to display data from multiple scales and uses a hierarchical model of the relative positions of all nucleotide atoms in the cell nucleus, i.e., the complete physical genome. Our model framework is flexible and adaptable to handle new more precise structural information as details emerge about the genome's physical arrangement. The large amounts of data generated by high-throughput or whole-genome experiments raise issues of scale, storage, interactivity and abstraction. Novel methods will be required to extract useful knowledge. *Genome3D *is an early step toward such new approaches.

## Implementation

*Genome3D *is a GUI-based C++ program which runs on Windows (XP or later) platforms. Its software architecture is based on the Model-Viewer-Controller pattern [11]. *Genome3D *is a viewer application to explore an underlying physical model displaying selections and annotations based on its current user settings. To support multiple resolutions and maintain a high level of interactivity, the model is designed using an object-oriented, hierarchical data architecture [12]. *Genome3D *loads the model incrementally as needed to support user requests. Once a model is loaded, *Genome3D *supports UCSC Genome Browser track annotations of the BED and WIG formats [1].

At highest detail, a model of the physical genome requires a 3D position (x, y, z) for each bp atom of the genome. The large amount of such data (3 × 10^9 ^bp × 20 atoms/bp × 3 positions × 4 bytes ~ 600 gigabytes for humans) is reduced by exploiting the data's hierarchical organization. We store three scales of data for each chromosome in compressed XML format. Atomic positions are computed on demand and not saved. This technique reduces the storage size for a human genome to ~1.5 gigabytes, resulting in more than 400× savings. There are several sample models available for download from the *Genome3D *project homepage. More information of our representative model and its data format can be found in Additional file 1.

## Results and Discussion

### *Genome3D *Program Features

**Figure 1:**
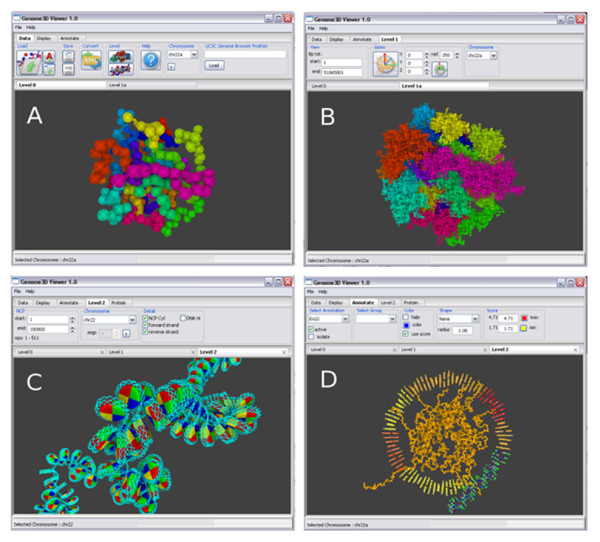
**The Genome3D application**. Four screen captures of *Genome3D *main windows showing progressive "drill-down" views of the same model of multi-resolution genomic data. All images were generated using a single instance of *Genome3D *and are differentiated solely by user-controlled display settings. **A **The lowest resolution is the nuclear scale and displays the steps of each giant loop random walk (see MM). **B **The 30 nm fiber scale of chromosomes corresponding to the giant loops shown in A. **C **At nucleosome resolution, a limited amount of DNA can be loaded and displayed. This image shows a segment of 100 K bp with approximate cylindrical NCPs and DNA strands represented as lines. **D **The highest resolution is the DNA scale which can resolve individual atoms. A single NCP is displayed here with bp-level annotations used to color each bp. Additionally, the image shows the atomic protein backbone structure of the NCP histones.

The range of scales and spatial organizations of DNA within the human cell presents many visualization challenges. To meet these challenges, *Genome3D *manipulates and displays genomic data at multiple resolutions. Figure 1 shows several screen captures of the *Genome3D *application at various levels of detail. Genome3D allows the user to specify the degree of detail to view, and the corresponding data is loaded dynamically. Because of the large amount of data and the limited memory that is available, only portions of the data can typically be viewed at high resolution. The interactivity of *Genome3D *facilitates exploring the model to find areas of interest. Additionally, the user can configure various display parameters (such as color and shape) to highlight significant structural relationships.

*Genome3D *features include:

• Display of genomic data from nuclear to atomic scale.

*Genome3D *has multiple windows to visualize the physical genome model from simultaneous different viewpoints and scales. The model resolution of the current viewing window is set by the user, and its viewing camera is controlled by the mouse. Resolutions and viewpoints depend of the type of data that is being visualized.

• A fully interactive point-and-select 3D environment

The user can navigate to an arbitrary region of interest by selecting a low resolution region and then loading corresponding higher resolution data which appears in another viewing window.

• Loading of multiple resolution user-created models with an open XML format

The *Genome3D *application adheres to the Model-View-Controller software design pattern [13]. The viewing software is completely separated from the multi-scale model that is being viewed. We have chosen a simple open format for each resolution of the model, and users can easily add their own models.

• Image capture and PovRay/PDB model export support

*Genome3D *supports screen capture of the current display image to a JPG format. For highly quality renders, it can export the current model and view as a PovRay model [14] format for off-line print quality rendering. In addition, atomic positions of selected DNA can be saved to a PDB format file for downstream analysis.

• Incorporation and user-defined visualization of UCSC annotation tracks onto the physical model

The UCSC Genome Database Browser has a variety of epigenetic information that can be exported directly from its web-site [1]. This data can be loaded into *Genome3D *and displayed on the currently loaded genome model.

### Visualizing Integrated Epigenetic and Genomic Data

We now give a few examples of applying biological information to a model and suggest possible methods of inferring unique structural relationships at various resolutions. One of the advantages of a multi-scale model is the ability to integrate data from various sources, and perhaps gain insight in higher level relationships or organizations. We choose to concentrate on high-throughput data sets that are becoming commonplace in current research: genome wide nucleosome positions, SNPs, histone methylations and gene expression profiles. The sample images, which can be visualized in *Genome3D*, were export and rendered in PovRay [14].

**Figure 2:**
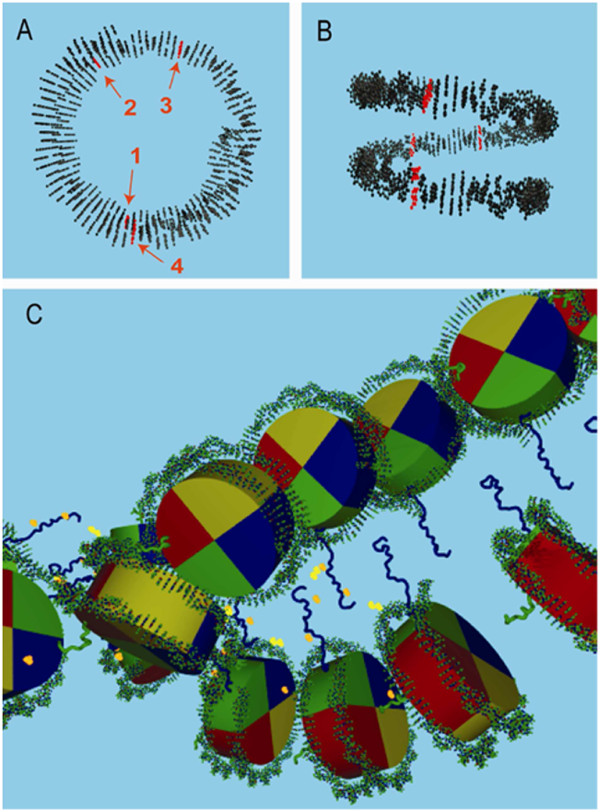
**Two examples of nucleosome epigenomic variation**. **A **Top view of 4 SNP variants rs6055249, rs7508868, rs6140378, and rs2064267 (numbered 1-4 respectively) within a non-coding histone of chromosome 20:7602872-7603018. The histone position was obtained from [18], the SNPs were taken from a recent study examining variants associated with HDL cholesterol [19]. Such images may reveal structural relationships between non-coding region SNPs and histone phasing. **B **Side view of A. **C **A series of histone trimethylations within ENCODE region ENr111 on chromosome 13:29668500-29671000 [27]. The histone bp positions are from [18]. Each histone protein is shown as an approximate cylinder wedge: H2A (yellow), H2B (red), H3 (blue), H4 (green). The CA backbones of the H3 and H4 N-terminal tails are modeled using the crystal structure of the NCP (PDB 1A0I) [28]. The bright yellow spheres indicate H3K4me3 and H3K9me3, and the orange spheres are H3K27me3, H3K36me3 and H3K79me3.

The impact of nucleosome position on gene regulation is well-known [15]. In addition to nucleosome restructuring/modification [16], the rotation and phasing information of DNA sequence may also play a significant role in gene regulation [17], particularly within non-coding regions. Figures 2A, B show a non-coding nucleosome with multiple SNPs using genome-wide histone positioning data [18] combined with a SNP dataset [19]. It highlights one of the advantages of three dimensional genomic data by clearly showing the phasing of the SNPs relative to the histone. Observations of this type and of more complicated structural relationships may provide insights for further analysis, and such hidden three-dimensional structure is perhaps best explored with the human eye using a physical model.

Another important source of epigenomic information is histone modification. Genome-wide histone modifications are being studied through a combination of DNA microarray and chromatin immunoprecipitation (ChIP-chip assays) [20]. Histone methylations have important gene regulation implications, and methylations have been shown to serve as binding platforms for transcription machinery. The ENCODE initiative [21] is creating high-resolution epigenetic information for ~1% of the human genome. Despite the fact that such modification occurs in histone proteins, current approaches to map and visualize such information are limited to sequence coordinates in the genome. Our physical genome model visualizes methylation of histone proteins at atomic detail as determined by crystal structure. Figure 2C shows histone methylations for several histones within an ENCODE region. An integrated physical genome model can show the interplay between histone modifications and other genomic data, such as SNPs, DNA methylation, the structure of gene, promoter and transcription machinery, etc.

**Figure 3:**
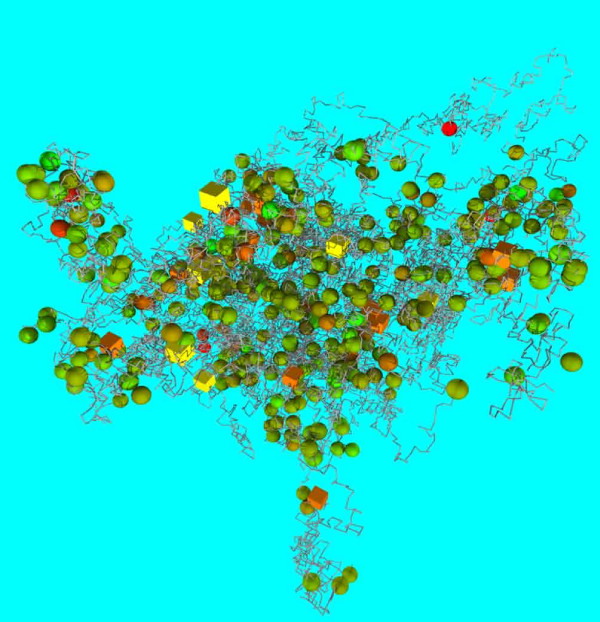
**A sample microarray expression data set superimposed on a chromosome model**. The figure integrates p53 knock-out expression data [23] and p53 binding site information within chromosome 19. It demonstrates how array data can be annotated on an existing physical model, and how large structural effects could be observed. The colored spheres show the magnitude of gene expression change when p53 is disrupted (green +/red -). Each sphere represents the transcription start site (TSS) of a gene. Cubes are placed at p53 binding sites, and are colored orange if close (< 10 kbp) to a measured gene expression TSS, yellow otherwise.

In addition to epigenomic data, the physical genome model also provides a platform to visualize high-throughput gene expression data and its interplay with global binding information of transcription factors. We consider a sample analysis of transcription factor P53. Genome-wide binding sites of P53 proteins [22] can be combined with the gene expression results from a study investigating the dosing effect of P53 [23]. This may identify genes that have P53 binding sites in their promoter regions and are responsive to the dosing effect of P53 protein. Such large-scale microarray expression data is often displayed with a two-dimensional array format, emphasizing shared expression between genes, while P53 binding data are stored in tabular form. With a physical model, expression levels of genes in response to P53 level can be mapped to genome positions together with global P53 binding information, revealing any structural bias of the expression. Figure 3 shows this type of physical genome annotation. Drawing inferences from coupling averaged or "snap-shot" expression data with the dynamic architecture of the genome may be helpful in determining structural dependences in expression patterns.

## Discussion

To illustrate the capability of *Genome3D *to integrate and examine data of appropriate scales, we constructed an elementary model of the physical genome (see Additional file 1 for details). This basic model is approximate since precise knowledge of the physical genome is largely unknown at present. However, the model's inaccuracies are secondary to its multi-scale approach that provides a framework to improve and refine the model. Current technologies are making significant progress toward capturing chromosome conformation within the nucleus at various scales [24,25]. Because our multi-scale model is purely descriptive beyond the NCP scale, it can easily incorporate more accurate structural folding information, such as the 'fractal globule' behaviour [26]. The *Genome3D *viewer, decoupled from the genome model, can be used to view any model that uses our model framework.

Building a 3D model of a complete physical genome is a non-trivial task. The structure and organization at a physical level is dynamic and heavily influenced by local and global constraints. A typical experiment may provide new data at a specific resolution or portion of the genome, and the integration of these data with other information to flesh out a multi-resolution model is challenging. For example, an experiment may measure local chromatin structure around a transcription site. This structure can be expressed as a collection of DNA strands, NCPs, and perhaps lower resolution 30 nm chromatin fibers. Our data formats are flexible enough to allow partial integration of this information, when the larger global structure is undetermined, or inferred by more global stochastic measurements from other experiments. Combining such data across resolutions is often difficult, but establishing data formats and visualization tools provide a framework that may simplify the integration process.

## Conclusions

Recent advances in determining chromosome folding principles [24] highlight the need for new visualization methods. More detailed three-dimensional genomic models will help in discovering and characterizing epigenetic processes. We have created a multi-scale genomic viewer, *Genome3D*, to display and investigate genomic and epigenomic information in a three-dimensional representation of the physical genome. The viewer software and its underlying data architecture are designed to handle the visualization and integration issues that are present when dealing with large amount of data at multiple resolutions. Our data structures can easily accommodate new advances in chromosome folding and organization.

A common framework of established scales and formats could vastly improve multi-scale data integration and the ability to infer previously unknown relationships within the composite data. Our model architecture defines clear demarcations between four scales (nuclear, fiber, nucleosome and DNA), which facilitates data integration in a consistent and well-behaved manner. As more data become available, the ability to model, characterize, visualize, and perhaps most crucially, integrate information at many scales is necessary to achieve fuller understanding of the human genome.

## Availability and Requirements

Project name: Genome3D

Project homepage: http://genomebioinfo.musc.edu/Genome3D/Index.html

Operating System: Windows-based operation systems (XP or later)

Programming Language: C++ and Python

Other requirements: OpenGLv2.0 and GLSL v2.0 (may not be present on some older graphics adapters - see Additional file 2)

Any restrictions to use by non-academics: None

## Authors' contributions

WJZ conceived the initial concept of the project and developed the project with TMA. TMA developed the 3D genomic model and worked with MM to develop the Genome3D software. JT and WJZ advised TMA and MM on the software development, and WJZ oversaw the whole project. All authors read and approved the final manuscript.

## Supplementary Material

Additional file 1 **Supplemental information**. Additional details about human physical genome model construction and the Genome3D software. Click here for file

Additional file 2 **Genome3D v1.0 README**. The README file for Genome3D software. Click here for file
