# Supplementary material for: Genome3D: A viewer-model framework for integrating and visualizing multi-scale epigenomic information within a three-dimensional genome
Source: BMC Bioinformatics. 2010 Sep 2;11:444. doi: 10.1186/1471-2105-11-444 (PMC2941692; doi:10.1186/1471-2105-11-444)
Supplement: Additional file 1 — Supplemental information. Additional details about human physical genome model construction and the Genome3D software. [file 1471-2105-11-444-S1.PDF]

Additional File 1: Supplemental Information

The Physical Genome Model

To explore typical visualization concerns, we created a physical model of the human genome in the G1 interphase. Interphase chromatin has limited condensation along with continual transcriptional activity, exhibiting maximal structural detail. Figure S1 displays a full synthetic model of human G1 chromatin at 30nm resolution, with select regions magnified at higher resolutions. Using characteristic statistics, we have generated 46 human chromosomes in a 3-dimensional space comparable in size to a human nucleus (~10 µm). Although our model is of course approximate, its size and scale are quite similar to what would be expected for more accurate models.

We chose to model four scales which capture the majority of function and organization of nuclear DNA: nuclear, 30nm, nucleosome, and atomic (listed in order of increasing resolution). The lower resolutions of our model, i.e., the 30nm chromatin and nuclear scales, contain data that possess a random element, and their statistical characteristic distributions are based on experimental observation [2-4]. Although the nuclear periphery is not explicitly modelled, the computed path of the 30nm chromatin is designed to meet nucleosome density and curvature statistics measured *in situ* [4]. A central feature of our model is its integration of multi-resolution data from different scales to realize a fully atomic model. Table S1 lists the scales and sources of the data sets used in constructing our model. Our model is modular and the

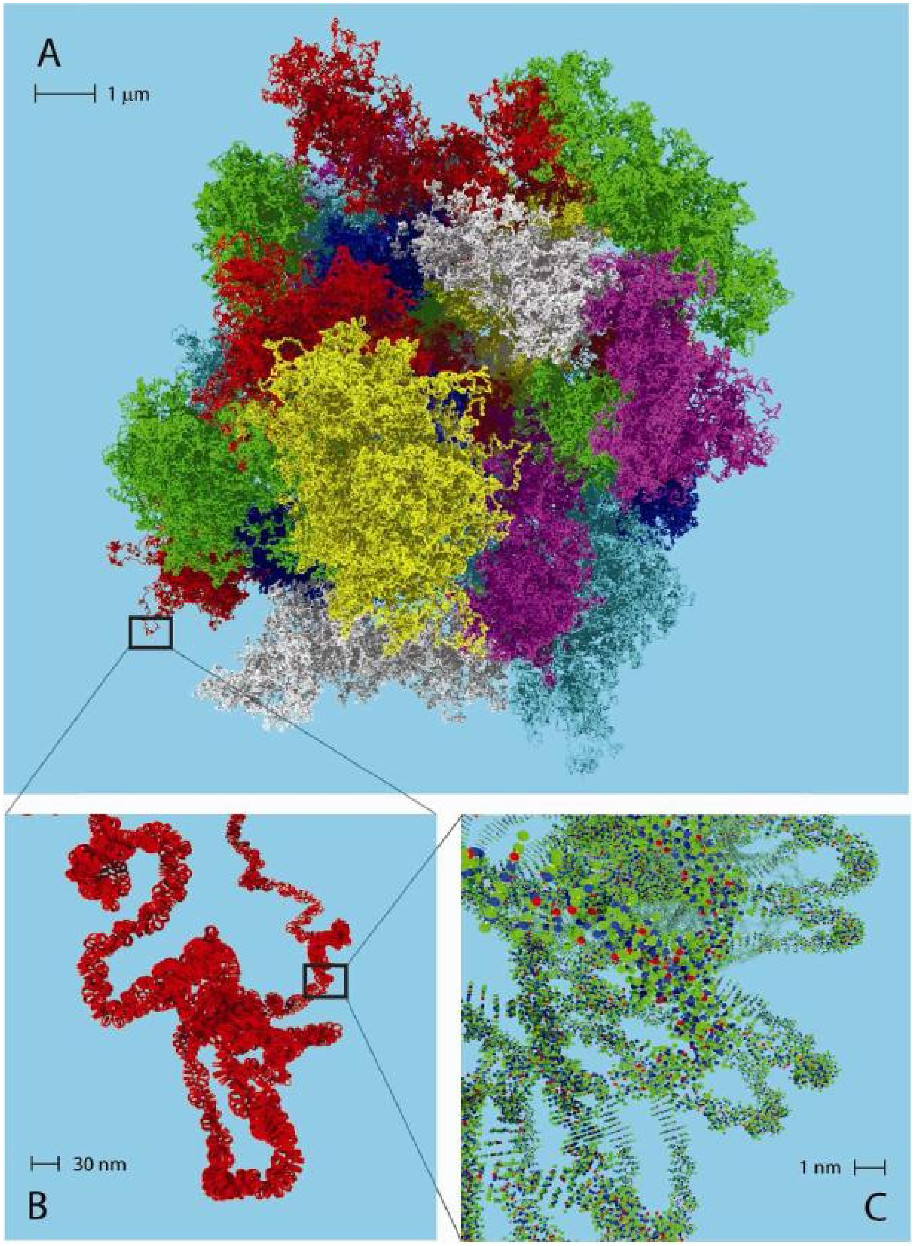

Figure S1. A multi-scale view of the human physical genome model

The model was generated for all 46 chromosomes in G1 interphase. **A** At nuclear scale, all chromosomes are shown as 30nm fibers in separate chromosome territories (CTs). Each chromosome was colored with one of eight random colors. The spatial distribution of chromosomes was derived from statistical methods. **B** A small portion of 30nm chromatin shown in A (bounded by black box) displayed at nucleosome resolution. At this scale, small loops are visible as DNA wraps around the nucleosome core particle (not shown). **C** An atomic view of a nucleosome region in B. The atoms are placed according to standard Watson-Crick base pairings [1]. A movie of this model is provided on the Genome3D web page (http://genomebioinfo.musc.edu/Genome3D/Index.html).

statistically derived data can be replaced by more detailed and accurate empirical spatial

distributions.

The levels are constructed in a top-down order, with each level using data from the next higher scale as starting positions. These anchor points constrain the lower scale structure and enforce a consistent overall model. This hierarchy efficiently stores data and allows for “drill-down” information retrieval. The only input data required for the model are the DNA sequence length of each chromosome. Atomic resolution data is generated on demand, and sequence data is only accessed when necessary by the viewer. If available, NCP bp positions, such as those from Schones et al [5], can be used. Because of the randomness present in the nuclear and chromatin scales, each build procedure creates a different instance of a physical genome. Values of the physical constants used are given in Table S2.

Data Structures

There are two basic geometric elements in our model which are used in multiple scales: the 3-dimensional curve and random walk. Three-dimensional curves are typically described by control points with some form of interpolation. We desire interpolations through control points to gain precise control of the curve position and prevent intersections. Catmull-Rom splines pass through their control points, but sacrifice C2 (curvature) continuity [6]. In our model, with DNA twist determined by bp position, we only require C1 (tangent) continuity, and thus use Catmull-Rom curves to describe genome curves at the fiber, nucleosome and DNA scales. Since each control point is associated with a unique bp, the curves are parameterized by bp index.

Random walks (RWs) can be simulated by imposing a cubic lattice on the walk space and using an integer hash-table to keep track of each successive position. During the building of the 30nm fiber GL RW phase, it is necessary for a RW of N steps to start and end in separately proscribed lattice cubes. This is an example of a constrained RW, and we use the following energy function to bias the choice of directions:

$$P(unconstrained) = \frac{\overline{N} - D_L}{\overline{N}},$$

where $\overline{N}$ is the number of steps remaining and D_L is the cube lattice distance to the goal. This function is an approximate measure of the "slack" in the curve. If this test fails, the choice is constrained to be a step in the direction of the goal.

Scale 1: Nuclear

We model interphase chromatin of the G1 phase only, which has a significant degree of unwinding due to high levels of transcription and less crowding because of single copy DNA material. Viewed at the scale of a cell nucleus (~5-15 µm), DNA is seen as clusters and is considered to be predominately localized with limited overlap [7] or with more substantial intermingling [8]. One chromatin model [9] is based on observing transcriptional activity on the surface of tightly integrated chromosome territories (CTs) and within inter-chromatin domains. Another model, known as the Giant Loop/ Random Walk (hereafter referred to as GL/RW, respectively) model [2] is built to match experimental intra-chromatin distance measures, and

transcription may occur anywhere. There is evidence for both models [10, 11], but we find the GL/RW model more descriptive of overall chromatin structure, and can support random degrees of intermingling.

We arbitrarily use chromosome size to establish relative positions within the nucleus by biasing the larger chromosomes toward the perimeter [3]. This semi-random sequence is the chromosome build order, starting from the nuclear center to the perimeter. Other chromosome arrangements, such as those dependent on cell type [12], are easily modelled at this level as different sequences. A GL RW is then constructed for each chromosome. These RWs outline the extent of each chromosome at low resolution, and the data is saved as control points.

Scale 2: Fiber (30nm)

A number of experiments have characterized the behavior of DNA at this level of compaction, where DNA is seen as individual fibers of approximately 30 nm widths when viewed by Electron Microscopy. *In vitro* experiments show that the fibers move as random coils with a persistence length of anywhere from 30-220 nm, depending on the species [4]. In the cell, there appear to be two major modes of random behavior seen at different fiber scales: the random coil behavior for lengths of < 2 Mbps; and an additional constraint which begins to restrict the random behaviour at larger lengths (on the order of 2-200 Mbps), This creates two structural hierarchies within the chromosome – one is based on a large scale restriction and another forms loops or inter-hub chromatin, which is the basic 30nm random coil.

At the 30nm scale, constrained RWs are performed for each step of the GLs computed at the nuclear scale. To a much larger extent than GL RWs, the 30nm RWs must contend with crowding, intersections and inter-twining. We address this issue by further constraining 30nm RWs: a) lattice cubes can only be occupied by one chromosome and b) a buffer radius of $2L_p$ is placed around established RW steps to avoid overlap with successive RWs. This limits inter-twining and increases CT integrity. Self-avoidance is modelled by allowing at most 2 visits to any lattice cube by a single RW.

The 30nm fiber RWs are created separately and are then translated toward the center of the nucleus until an intersection with another chromosome occurs. This shrinking process is repeated from random directions and orientations to maximize nuclear packing, and creates inter-chromosomal distances of ~5nm. Depending on the shape of the non-convex chromosomes, intermingling volumes can result. Although we do not explicitly model containment or compression forces from the nuclear envelope, the GL RWs constrict the 30nm fiber in a manner consistent with chromosome nuclear density.

Scale 3: Nucleosome

The nucleosome level encompasses the transition from linear DNA to the 30nm fiber.
There are several models which attempt to explain the compaction of DNA via histones to the 30nm fiber. Most models fall into two classes: one-start helix (solenoid [13], inter-digitated solenoid [14]) and two-start helix (zigzag helical ribbon [15], double-helical cross-linker [16]).

Both types of models can give similar DNA concentrations [17] and allow for variation and irregularities.

The recurring structural motif at this scale is known as the “nucleosomal repeat”, which includes the nucleosome core particle (NCP) [18] and the linker DNA that has been found to vary between 167-250 bp [19]. This implies that the linker region varies from 20 to 80 bp, as the NCP is a highly conserved structural unit 147 bp in length. We model the nucleosome level using a recent high-resolution nucleosome array crystal structure [20] as the nucleosomal repeat.

The nucleosomal repeat structure is measured against a fiber axis, which indicates the relative orientation of the NCPs to the 30nm fiber as they precess around the axis in a two-start manner. We first propagate the nucleosomal repeat unit forward to be consistent with the crystal structure, but then translate and orient it to match the sampled orientation from the 30nm fiber.

The regularity of the NCP can be used to reduce the amount of data required to characterize the DNA atomic chain. The chain forms a helix around each NCP with a constant radius and rise [21]. By representing the DNA chain as a single 3d curve with ~1nm cross-section [22], a single starting bp index, position and orientation of each NCP is sufficient to derive atomic positions. Starting NCP bp indices were derived from a recent nucleosome position data set [5] and vacancies were filled with the standard NCP and linker.

Scale 4: DNA (atomic)

The atomic level of detail allows integration high-resolution epigenomic data, such as DNA or histone methylations. In addition, it provides a platform for characterizing protein-protein and protein-DNA atomic interactions, which play important roles in gene regulation. We also show how crystal structures can augment the model by using PDB data of a tetra-nucleosome array[20] and the nucleosome core particle [18]. High resolution detail for the genome model increases its capacity to incorporate and refine new structural information in the global context of entire genome.

The atomic nature of linear double-stranded DNA (dsDNA) has been well characterized and is highly regular. Therefore, standardized ideal planar Watson-Crick base pairs of B-form DNA [1] are sufficient to model at this level. We are not attempting to capture alternative motifs or non-uniformities based on sequence at this stage [23]. Evaluating the nucleosome scale curve returns a tangent vector at each base pair index. A curvature vector is computed by accumulating the total DNA twist offset from the first base pair. This accumulation can be explicitly determined by assuming constant DNA twist values [21] within the nucleosomal repeat unit.

Input Data Formats

Genome3D reads model information from data files in the resolutions: Giant Loop, Fiber and Nucleosome Level. Each format initially stored in XML data. The Fiber and Nucleosome Level data are converted to a binary format for faster loading. Full genome sample models are available for download here .

Genome3D uses a multi-resolution model stored in three resolutions in separate folders. A single chromosome has information contained in an XML file in each resolution directory:

1. gloop /

   - low resolution curves control points

   - each curve is a "giant loop" the anchors the chromosome

   - stored as an XML curve

2. fiber /

   - medium resolution curves control points

   - each curve is a path of 30nm chromatin for each chromosome

   - stored as an XML curve (usually in binary to conserve space and speed up load time)

3. nucleo /

   - high resolution data

   - nucleosome core particles positions and orientations for each chromosome

   - stored as an XML NCP file (usually in binary to conserve space and speed up load time)

In practice, it will often be difficult to obtain experimental data for all 3 levels, and some amount of integration will have to be performed. This is a non-trivial task to make the data consistent across resolutions. We presented above a deterministic method to flesh out an atomic model from a given 3d curve at the fiber (30nm) resolution, but this may be insufficient for some cases where the input data is at higher resolution. The local and global chromatin structure may be difficult to reconcile, most likely from lack of data at the global level. In these cases, partial model building may be helpful, as the input data formats need not be complete chromosomes.

XML curve

This file format is a simple 3d curve defined by its control points. The curve is parameterized by base pair index. We used a Catmull Rom spline to interpolate the positions as it passes thru control points. Since 4 points are need to interpolate, 2 points were added at the front and back to permit interpolation over the full range of the curve. The additional points were computed by reflecting the end regions. To prevent the bp from being negative, the bp index is offset by 10000, that is, to get the position of bp 1 from the curve, you interpolate bp 10001 (which uses 2 virtual reflected bps which have an index < 1000 but > 0)

```
<curve>
    <description>
        <name>chr1<name>
        <time>Mon Mar 17 16:59:02 2008<time>
        <num_keys>49744<num_keys>            <-- number of control points
(keys ) -->
    <description>
    <keys>
        <key bp="10001">                            <-- bp index --->
            <x>-1621.519362<x>             <-- control point x coord -->
            <y>-4515.041581<y>             <-- control point y coord -->
            <z>1233.750178<z>              <-- control point z coord -->
        <key>
```

```
        ....
    <keys>
<curve>
```

NOTE: all distances are in nanometers (nm).

There is no requirement that the curve by any set length, just that the number of keys are completely specified. Thus, you can create curves of any bp index starting and ending points in any order. This is useful if you are modeling a portion of the genome.

XML NCP

This file format is a list of ncps (Nucleosome Core Particle) within the chromosome. Each ncp is 147 bps long spiral, with a radius and rise, and has a an unique position and orientation define by a radial vector and an axis. We store a ncp with 3 vectors, the center vector, the radial vector, and the axis vector. All other parameters are assumed to be constant and are in the header. Like XML curves described above, the number of ncps in an XML nucleo file is arbitrary and not dependent on file order.

The description composite gives fixed information about each ncp. DNA wraps around the ncp in a spiral. The total rotation is give in the rot rad tag in radians (it's 1.75 turns). The rise is the amount for rise per turn for each revolution. i.e. the slope of the spiral.

Each NCP is specified by 3 vectors, which are all specified with respect to the global nuclear frame.

1. center (**C**)

   The center of the NCP rotation. Note that this is *not* the center of mass.

2. radial (**R**)

This vector is the distance and direction from the center to the first bp of the NCP. Note that this is a relative vector, to get the position of the first bp, compute **C** + **R**.

3. axis (**A**)

   A unit vector pointing in the direction of the axis of rotation of the NCP helix.

To create a DNA spiral around ncp, spin **R** about **A** centered at **C** for rot_rad radians, bumping the position up the axis by rise_nm each turn. (see Figure S2)

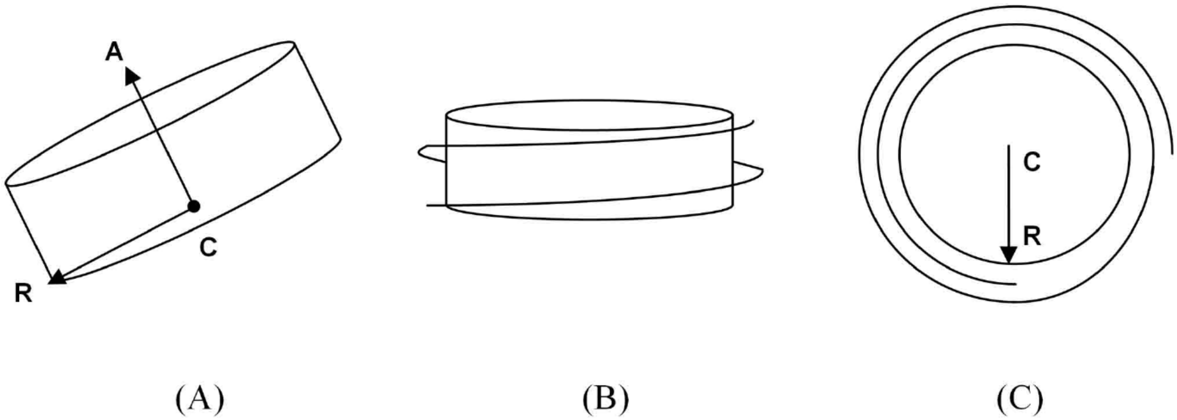

Figure S2 – A diagram of NCP (Nucleosome Core Particle) orientation vectors. (A) The position of the C (center), A (axis of rotation) and R(radius to starting bp) vectors. (B) A side view of the NCP showing the DNA helix circumscribing the NCP. The pitch of this helix is rise_nm/rot_rad. (C) A view looking down the axis of rotation. The DNA helix completes 1.75 turns about the NCP.

A sample NCP in an XML NCP file:

```
<nucleo>
    <description>
        <name>chr1<name>
        <time>Wed May  7 23:02:08 2008<time>
        <rise_nm>2.600000<rise_nm>     <-- rise per turn in nm -->
        <rot_rad>10.995574<rot_rad>    <-- length of dna spiral around ncp in
radians -->
        <ncp_bps>146<ncp_bps>          <-- number of bps in dna ncp wrap -->
        <num_ncps>1006639<num_ncps>    <-- number of ncps in file -->
    <description>
    <ncps>
```

```
        <ncp bp="1" helix="A" data="none">     <-- bp is bp index
   the following attributes are annotations and do not affect structure
   helix = which part of the 2-start helix - either A or B
   data = position source annotation   -->
            <cx>-1623.008126<cx>       <-- center of ncp x coord -->
            <cy>-4513.744538<cy>       <-- center of ncp y coord -->
            <cz>1240.902401<cz>        <-- center of ncp z coord -->
            <ax>-0.300901<ax>          <-- axis of ncp x coord -->
            <ay>0.908627<ay>           <-- axis of ncp y coord -->
            <az>-0.289578<az>          <-- axis of ncp z coord -->
            <rx>2.386502<rx>           <-- radius of ncp x coord -->
            <ry>-0.418208<ry>          <-- radius of ncp y coord -->
            <rz>-3.792059<rz>          <-- radius of ncp z coord -->
        <ncp>
        ....
    <ncps>
<nucleo>
```

NOTE: all distances are in nanometers (nm).

For rendering purposes, a local reference frame for each NCP is established. It is based on the center of the NCP and the radial vector. The radial vector is considered to be the X axis, the NCP axis of rotation is the Y axis and the Z axis is orthogonal to two. This is the coordinate system in which all proteins are rendered.

Tables

Table S1 - A list of the data sets used in modelling and creating images of the human physical genome.

Integrated Datasets		
Type	*scale*	*source*
Genome sequence	atomic	UCSC hg18 build [24]
Chromosome positions*	nuclear	FISH study [3]
30 nm fiber path/ flexibility*	30nm	Persistence length[25]
Nucleosome bp positions	nucleo	MNase-digested chromatin [5]
Nucleosome core particle	atomic	PDB 1AOI[18]
Tetra-nucleosome array	atomic	PDB 1ZBB[20]
Single nucleotide polymorphisms	atomic	FUSION, SardiNIA, DGI databases [26]
Histone methylations	atomic	ENCODE Region ENm010[27]
Bp atom positions	atomic	NC-IUBMB standard reference frame [1]

The data spans four resolutions: nuclear (~1µm), 30nm, nucleosomal (~1nm) and atomic (~1Å). It consists of statistical information, atomic crystal structures and high-throughput genome-wide data. The ability to incorporate data from different resolutions is vital to creating a consistent model. Information labelled with * is statistical in nature and introduces a random element to the model.

The above table show the resolution span of information that has been used to create our sample models. It is by no means comprehensive and is provided as an example of how data from different scales is to be integrated to create a single multi-resolution model. High resolution atomic data can often be considered uniform given lower resolution constraints, and we designed our model top-down, where each proceeding level established constraints for the next higher resolution. Obtaining more precise data, particularly at the 30nm chromatin level, is difficult currently, and we use global statistics to provide plausible local chromatin structure. Complicated inter-resolution interactions, such as large sequence-based interactions or

transcriptional crhomatin remodeling, provide more constraints and will require more complicated model-building paradigms.

Table S2 - Physical constants used in build the genomic model

Physical Constants	
Nuclear scale	
Nuclear radius	15µm
Giant Loop *Lp*	300nm [2]
Giant Loop *Lp* bp	3000000 [2]
30nm Fiber scale	
30nm *Lp* [25]	50nm
30nm *Lp* bp [25]	5000
Nucleosomes scale	
NCP diameter	9nm [21]
NCP rise	2.59nm/turn [21]
NCP bp	147bp [21]
NRU fiber rise	3.34nm/NRU [20]
NRU fiber twist	-38.1°/NRU [20]
DNA scale	
DNA twist	10.5 bp/turn [21]
DNA NCP twist	10.2 bp/turn [21]

L_p is persistence length. NCP is the nucleosomal core particle. NRU is the nucleosome repeat unit. These values are reasonable estimates from current literature.

References

1. Olson WK, Bansal M, Burley SK, Dickerson RE, Gerstein M, Harvey SC, Heinemann U, Lu XJ, Neidle S, Shakked Z, Sklenar H, Suzuki M, Tung CS, Westhof E, Wolberger C, Berman HM: **A standard reference frame for the description of nucleic acid base-pair geometry**. *J Mol Biol* 2001, 313(1):229-237.
2. Sachs RK, van den Engh G, Trask B, Yokota H, Hearst JE: **A random-walk/giant-loop model for interphase chromosomes**. *Proc Natl Acad Sci U S A* 1995, 92(7):2710-2714.
3. Sun HB, Shen J, Yokota H: **Size-dependent positioning of human chromosomes in interphase nuclei**. *Biophysical journal* 2000, 79(1):184-190.
4. Bystricky K, Heun P, Gehlen L, Langowski J, Gasser SM: **Long-range compaction and flexibility of interphase chromatin in budding yeast analyzed by high-resolution imaging techniques**. *Proc Natl Acad Sci U S A* 2004, 101(47):16495-16500.
5. Schones DE, Cui K, Cuddapah S, Roh TY, Barski A, Wang Z, Wei G, Zhao K: **Dynamic regulation of nucleosome positioning in the human genome**. *Cell* 2008, 132(5):887-898.
6. Foley JD: **Computer graphics : principles and practice**, 2nd edn. Reading, Mass.: Addison-Wesley; 1995.
7. Cremer T, Cremer C: **Chromosome territories, nuclear architecture and gene regulation in mammalian cells**. *Nat Rev Genet* 2001, 2(4):292-301.
8. Branco MR, Pombo A: **Intermingling of chromosome territories in interphase suggests role in translocations and transcription-dependent associations**. *PLoS Biol* 2006, 4(5):e138.
9. Zirbel RM, Mathieu UR, Kurz A, Cremer T, Lichter P: **Evidence for a nuclear compartment of transcription and splicing located at chromosome domain boundaries**. *Chromosome Res* 1993, 1(2):93-106.
10. Cremer T, Cremer M, Dietzel S, Muller S, Solovei I, Fakan S: **Chromosome territories--a functional nuclear landscape**. *Current opinion in cell biology* 2006, 18(3):307-316.
11. Konig P, Braunfeld MB, Sedat JW, Agard DA: **The three-dimensional structure of in vitro reconstituted Xenopus laevis chromosomes by EM tomography**. *Chromosoma* 2007, 116(4):349-372.
12. Tanabe H, Muller S, Neusser M, von Hase J, Calcagno E, Cremer M, Solovei I, Cremer C, Cremer T: **Evolutionary conservation of chromosome territory arrangements in cell nuclei from higher primates**. *Proc Natl Acad Sci U S A* 2002, 99(7):4424-4429.
13. Smith MF, Athey BD, Williams SP, Langmore JP: **Radial density distribution of chromatin: evidence that chromatin fibers have solid centers**. *The Journal of cell biology* 1990, 110(2):245-254.
14. Robinson PJ, Fairall L, Huynh VA, Rhodes D: **EM measurements define the dimensions of the "30-nm" chromatin fiber: evidence for a compact, interdigitated structure**. *Proc Natl Acad Sci U S A* 2006, 103(17):6506-6511.

15. Woodcock CL, Frado LL, Rattner JB: **The higher-order structure of chromatin: evidence for a helical ribbon arrangement**. *The Journal of cell biology* 1984, 99(1 Pt 1):42-52.
16. Williams SP, Athey BD, Muglia LJ, Schappe RS, Gough AH, Langmore JP: **Chromatin fibers are left-handed double helices with diameter and mass per unit length that depend on linker length**. *Biophysical journal* 1986, 49(1):233-248.
17. Daban JR: **Physical constraints in the condensation of eukaryotic chromosomes. Local concentration of DNA versus linear packing ratio in higher order chromatin structures**. *Biochemistry* 2000, 39(14):3861-3866.
18. Luger K, Mader AW, Richmond RK, Sargent DF, Richmond TJ: **Crystal structure of the nucleosome core particle at 2.8 A resolution**. *Nature* 1997, 389(6648):251-260.
19. Sen D, Mitra S, Crothers DM: **Higher order structure of chromatin: evidence from photochemically detected linear dichroism**. *Biochemistry* 1986, 25(11):3441-3447.
20. Schalch T, Duda S, Sargent DF, Richmond TJ: **X-ray structure of a tetranucleosome and its implications for the chromatin fibre**. *Nature* 2005, 436(7047):138-141.
21. Richmond TJ, Davey CA: **The structure of DNA in the nucleosome core**. *Nature* 2003, 423(6936):145-150.
22. Barbi M, Mozziconacci J, Victor JM: **How the chromatin fiber deals with topological constraints**. *Phys Rev E Stat Nonlin Soft Matter Phys* 2005, 71(3 Pt 1):031910.
23. Lu XJ, Olson WK: **3DNA: a software package for the analysis, rebuilding and visualization of three-dimensional nucleic acid structures**. *Nucleic Acids Res* 2003, 31(17):5108-5121.
24. Karolchik D, Kuhn RM, Baertsch R, Barber GP, Clawson H, Diekhans M, Giardine B, Harte RA, Hinrichs AS, Hsu F, Kober KM, Miller W, Pedersen JS, Pohl A, Raney BJ, Rhead B, Rosenbloom KR, Smith KE, Stanke M, Thakkapallayil A, Trumbower H, Wang T, Zweig AS, Haussler D, Kent WJ: **The UCSC Genome Browser Database: 2008 update**. *Nucleic Acids Res* 2008, 36(Database issue):D773-779.
25. Ponomarev AL, Brenner D, Hlatky LR, Sachs RK: **A polymer, random walk model for the size-distribution of large DNA fragments after high linear energy transfer radiation**. *Radiation and environmental biophysics* 2000, 39(2):111-120.
26. Willer CJ, Sanna S, Jackson AU, Scuteri A, Bonnycastle LL, Clarke R, Heath SC, Timpson NJ, Najjar SS, Stringham HM *et al*: **Newly identified loci that influence lipid concentrations and risk of coronary artery disease**. *Nat Genet* 2008, 40(2):161-169.
27. **The ENCODE (ENCyclopedia Of DNA Elements) Project**. *Science* 2004, 306(5696):636-640.
