# Supplementary material for: Genome3D: A viewer-model framework for integrating and visualizing multi-scale epigenomic information within a three-dimensional genome
Source: BMC Bioinformatics. 2010 Sep 2;11:444. doi: 10.1186/1471-2105-11-444 (PMC2941692; doi:10.1186/1471-2105-11-444)
Supplement: Additional file 2 — Genome3D v1.0 README. The README file for Genome3D software. [file 1471-2105-11-444-S2.HTML]

REAdME

  

Genome3D v1.0
README

  
Genome3D is a genome visualization program written for Windows-based
operation systems ( XP and later ). It can be downloaded from http://genomebioinfo.musc.edu/Genome3D/Index.html.  
  
  
Installing Genome3D  
  
The file Genome3D\_Viewer\_with\_Examples.zip contains two folders:  
  
Genome3D\_Viewer/  
Genome3D\_Examples/  
  
Unzip this file to a local program directory. The executable and all support
files necessary to run it are contained in the folder Genome3D\_Viewer/. Open
this folder, and double-click on the executable file named "Genome3D.exe". This
will start the applcation.  
  
It make by useful to create a Windows Shortcut from your Desktop to this file
to faciliate startup.  
  
Once the program is running, click on the "?" button in the application to view
the documentation. The Help Documentation is located in the
Genome3D\_Viewer/helpDir/ folder.  
  
A good place to start is to load the examples or tutorials - see
below.  
  
  
Rendering
Requirements  
  
Genome3D uses OpenGL and GLSL to implement a SSAO (Screen Space Ambient
Occlusion) shader. This requires the OpenGL Frame Buffer Object(FBO) extension
and on-board memory to compile the shaders. These requirements may not be
present on some older graphics adapters.  
  
The application will not run if OpenGL v2.0 is not available. OPenGL v2.0 was
released in 2004. Please go here to read more and download if needed: http://www.opengl.org/.  
  
If your graphics adapter does not support the shader, the application will
still run, but will default to a simple lighting model resulting in inferior
quality images. However, in many cases, the shader functionality can be
obtained by loading a more recent device driver. Please consult your graphic
adapter vendor's web page for more.   
  
Additionally, there is a startup log file ("g3d.log") which is printed out in
the exectuable directory everytime the probgram executes. It contains debugging
information for the rendering pipeline at startup and may be useful.  
  
  
Examples  
  
Five example state files with demo models are located in the
Genome3D\_Examples/ folder. These files are provided to show the full range of
available data that can be visualized using Genome3D.   
  
These examples contain sample models that are not designed for in-depth
examination, they are partial models which show specific regions of the genome.
A full genome model is required to navigate without constraint. Full models can
be downloaded separately from http://genomebioinfo.musc.edu/Genome3D/Index.html.  
  
Details for each example is provided in the HTML Help
documentation.

Tutorials

There are three tutorials which provide step-by-step procedures to load,
examine and save the state of a single chromosome model. Unlike the examples,
which are saved states of previously annotated Genome3D views, the tutorials
work from a clean start and describe the steps to build a state than
demonstrates a particular aspect of the Genome3D's viewing capablilitles.
  
Details for each tutorial are provided in the HTML Help
Documentation.

Full
Models  
  
Two genome models are provided: Model A and B.  While both Model A and B
shares the same 3D configuration of their chromosomes in space, the nucleosome
position in these two models are different.  In Model A, the nucleosome
positions are determined by experimental approach (courtesy of Dr. K. Zhao from
NHLBI).  See Schones
DE, Cui K, Cuddapah S, Roh TY, Barski A, Wang Z, Wei G, Zhao K: Dynamic
regulation of nucleosome positioning in the human genome. Cell 2008,
132(5):887-898 for how the data are generated.  Model B has standard random
positioning of nucleosome.  Both models are provided in binary format at the
fiber and the nucleo level for small file size and fast load.    
  
Additional data are provided on the Genome3D web
page.  These include Model\_A and Model\_B in xml format. 
.  Two alternative
models, Genome3D\_Model\_C and Genome3D\_Model\_D, are also provided in xml
format.  In these two alternative models, the coordinates of nucleotides are
different from model A and B as a result of random walking algorithm.  
  
  
Genome Sequence File  
  
Chromosome sequences in FASTA format are loaded as required at
run-time.  All the models are built using human genome
assembly build 18 (hg18), which can be downloaded through UCSC genome
browser.  For example, to load the genome sequence of Chromosome 1 or
Chromosome 1a to the Genome3D viewer, download chr1.fa.gz, decompress the
downloaded file and point the viewer to the decompressed file.  If no
chromosome sequence is provided at run-time, ACGT will be used as default
instead.  
  
  
Documentation  
  
The Genome3D documentation is written in HTML and contained in helpDir/ in
the Genome3D\_Viewer/ folder. It is basic XML and can be viewed either from
within the application by pressing the "?" button, or using a separate Web
browser.  
  
  
Contact
Information  
  
Please address any questions or comments to:  
zhengw@musc.edu  
  
---------------  
  
 
